# Supplementary figures and images for: Combined Toxicity of the Most Common Indoor Aspergilli
Source: Pathogens. 2023 Mar 14;12(3):459. doi: 10.3390/pathogens12030459 (PMC10058518; doi:10.3390/pathogens12030459)

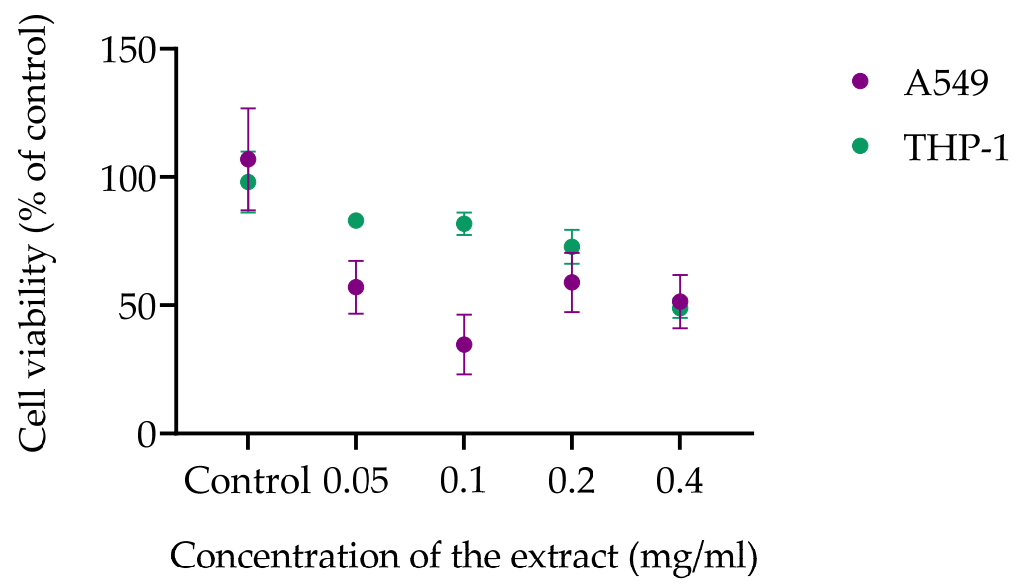

**Figure S1.** Cytotoxicity of extracted *A. protuberus* on A549 cells and THP-1 macrophages.

Supplement: Supplementary file 1 [file pathogens-12-00459-s001.zip › pathogens-2227427-supplementary.pdf]
